# Growth Inhibitory Effect of Polyunsaturated Fatty Acids (PUFAs) on Colon Cancer Cells via Their Growth Inhibitory Metabolites and Fatty Acid Composition Changes

**DOI:** 10.1371/journal.pone.0123256

**Published:** 2015-04-17

**Authors:** Chengcheng Zhang, Haining Yu, Xiaofeng Ni, Shengrong Shen, Undurti N. Das

**Affiliations:** 1 College of Biosystems Engineering and Food Science, Zhejiang University, Hangzhou, 310058, PR China; 2 College of Pharmaceutical Sciences, Zhejiang University of Technology, Hangzhou 310032, China; 3 UND Life Sciences, 2020 S 360^th^ St, #K-202, Federal Way, WA, 98003, United States of America; 4 Department of Medicine and BioScience Research Centre, GVP Hospital, GVP College of Engineering campus, Visakhapatnam-530 048, India; University of Nebraska Medical Center, UNITED STATES

## Abstract

**Background:**

Colorectal cancer is common. Polyunsaturated fatty acids (PUFAs) exert growth-inhibitory and pro-apoptotic effects on colon cancer cells. Metabolites of PUFAs such as prostaglandins (PGs), leukotrienes (LTs) and lipoxins (LXs) play a significant role in colon cancer.

**Methods:**

Human colon cancer LoVo and RKO cells were cultured with different concentration of PUFAs and 5-fluorouracil (5-FU) *in vitro*. Cell morphological changes, fatty acid composition, formation of PGE2, LTB4 and LXA4 and expression of COX-2, ALOX5, PGD synthase (PGDS), microsomal prostaglandin E synthase (mPGES) were assessed in LoVo and RKO cells when supplemented with PUFAs and 5-FU.

**Results:**

PUFAs and 5-FU inhibited growth of LoVo and RKO cells to the same extent at the doses used and produced significant alterations in their shape. As expected, higher concentrations of supplemented PUFAs were noted in the cells compared to control. LA, GLA, AA, ALA and EPA supplementation to LoVo cells suppressed production of PGE_2_, LTB_4_,and ALOX5, mPGES expression, but enhanced that of LXA_4_; whereas DHA enhanced PGE_2_ and LXA_4_ synthesis but decreased LTB_4_ formation and COX-2, ALOX5, mPGES expression. In contrast, 5-FU enhanced formation of PGE_2_, LTB_4_ and mPGES expression, but suppressed LXA_4_ synthesis and COX-2 expression. PGE_2_, LTB_4_ synthesis and ALOX5 expression was suppressed by LA, GLA, ALA and DHA; whereas AA, EPA and 5-FU enhanced PGE_2_ but paradoxically AA decreased and EPA and 5-FU enhanced LTB4 synthesis in RKO cells. All the PUFAs tested enhanced, while 5-FU decreased LXA_4_ formation in RKO cells; whereas GLA, AA, and 5-FU augmented while LA, ALA, EPA and DHA enhanced COX-2 expression in RKO cells.

**Conclusions:**

Tumoricidal action of PUFAs on colorectal LoVo and RKO cancer cells *in vitro* was associated with increased formation of LXA_4_, decreased synthesis of PGE_2_ and LTB_4_ and suppressed expression of COX-2, ALOX5, mPGES, whereas 5-FU produced contrasting actions on these indices.

## Introduction

Colorectal cancer is common both in males and females, with the highest incidence in Australia and New Zealand, Europe, and North America [1]. Western diet is rich in saturated fats and contains insufficient amounts of total polyunsaturated fatty acids (PUFAs) with the ratio between n-3 and n-6 being ~ 1: 20 that is considered to promote development of colorectal cancer. Previously, we and others showed that PUFAs (LA, GLA, AA, ALA, EPA and DHA) have inhibitory effect on the growth of tumor cells with little or no cytotoxic action on normal cells [2–5].

LA (linoleic acid = 9-cis,12-cis-octadecadienoic acid; 18:2 n-6) and ALA (α-linolenic acid = 9-cis,12-cis,15-cis-octadecatrienoic acid; 18:3 n-3) are essential fatty acids (EFAs) that form precursors to their respective long chain metabolites namely γ-linolenic acid (GLA; 18:3 n-6), dihomo-γ-linolenic acid (DGLA; 20:3 n-6) and arachidonic acid (AA, 20:4 n-6) and eicosapentaenoic acid (EPA, 20% n-3) and docosahexaenoic acid (DHA, 22:6 n-3) respectively. It is believed that Δ^6^ and Δ^5^ desaturases and respective elongases convert LA and ALA to their respective long-chain metabolites [6]. Tumor cells are known to be deficient in PUFAs, especially in AA, EPA and DHA due to decreased activity of enzymes Δ^6^ and Δ^5^ desaturases [7]. It may be noted that DGLA, AA and EPA form precursors to various prostaglandins (PGs), thromboxanes (TXs) and leukotrienes (LTs), whereas AA, EPA and DHA form precursors to lipoxins (LXs) (from AA), resolvins (from EPA and DHA) and protectins and maresins (from DHA) which play a significant role in inflammation and cancer [8]. In general, PGs, TXs, and LTs are pro-inflammatory in nature, produce immunosuppression and promote tumor cell proliferation. LXs, resolvins, protectins and maresins have potent anti-inflammatory actions [9, 10], but their exact role in the proliferation of tumor cells is not well documented though some studies did suggest that LXs may suppress their growth [6–8]. Although, it is believed that enhanced formation of various PGs, LTs and TXS from various PUFAs could be responsible for the cytotoxic action of PUFAs, there is no general consensus on this due to controversial results reported [6–8]. In contrast to the cytotoxic actions of PUFAs on tumor cells, some of these fatty acids, especially, GLA, PGE_1_ and PGI_2_ have been shown to possess cytoprotective and genoprotective actions [11–13]. On the other hand, overexpression of cyclooxygenase-2 (COX-2), which leads to the formation of excess of PGE_2_, has been associated with pro-inflammatory events and higher incidence of colorectal cancer [14–16]. These evidences indicate that COX-2 is a potential target for anti-cancer treatment. This is supported by the observation that n-3 PUFAs: DHA and EPA in combination with radiotherapy suppressed the growth of HT29 colon cancer cells that was associated with decreased COX-2 expression [17]. In addition, the microsomal PGE2 synthase (mPGES) is functionally linked to COX-2, which mediates the final regulatory step of PGE_2_ biosynthesis and are overexpressed in various cancers [18, 19].

In the present study, we evaluated the effect of various PUFAs (LA, GLA, AA of n-6 series and ALA, EPA, DHA of n-3 series) on the growth of colon cancer LoVo and RKO cells *in vitro* and compared these results with the known anti-cancer drug 5-fluorouracil (5-FU). In addition, we studied the effect of various PUFAs and 5-FU on alterations in the fatty acid composition, formation of PGE_2_, LTB_4_ and LXA_4_ and the expression of COX-2 that have not been reported previously.

## Materials and Methods

### Materials

The colorectal cancer cell lines, LoVo (undifferentiated) and RKO (semi-differentiated) were obtained from the Institute of Biochemistry and Cell Biology Shanghai Institute for Biological Sciences, Chinese Academy of Sciences. ALA, EPA, DHA, LA, GLA, AA and 5-FU were purchased from Sigma (St. Louis, MO, USA). RPMI1640 medium were purchased from GIBCO (Grand Island, NY, USA).

### Cell culture

LoVo and RKO cells were cultured in RPMI Medium1640 (GIBCO) supplemented with 10% fetal bovine serum, penicillin (100 U/ml), streptomycin (100 U/ml) and grown in a 5% CO2 humidified incubator at 37°C. The stock solutions of ALA, EPA, DHA, LA, GLA, and AA were prepared as described previously [20]. However, 5-FU was dissolved in high purity water as stock solution with the concentration of 50 mM. The stock solutions of fatty acids and 5-FU were filter-sterilized and freshly diluted with cell culture media when used. In all the studies, the doses of fatty acids and 5-FU used for LoVo were as follows: 5-FU 10μM, ALA 150 μM/ml, DHA 150 μM/ml, EPA 150 μM/ml, LA 150 μM/ml, AA 150 μM/ml and GLA 300 μM/ml and the cell density used was:1×10^5^ cells/ml; while the density of RKO was 5×10^4^ cells/ml and the dose of 5-FU, ALA, DHA, EPA, LA, AA, and GLA used were 0.4μM, 140μM/ml, 120μM/ml, 120μM/ml, 120μM/ml, 120μM/ml, 200μM/ml respectively.

### MTT assay and cell morphology determination

MTT (3-(4,5-dimethylthiazol-2-yl)-2,5-diphenylte-trazolium bromide (Sigma, USA)) assay was used to determine the effect of ALA, EPA, DHA, LA, GLA, AA and 5-FU on the proliferation of LoVo and RKO cells. MTT assay was performed as described previously [21].

LoVo cells were seeded in 24-well plates with a volume of 1 mL at a density of 1×10^5^ cells/ml, while the density of RKO was 5×10^4^ cells/ml. 24 hours after seeding, the cells were supplemented with different doses of various fatty acids and 5-FU for 48h. At the end of incubation, cell morphological changes were observed using an inverted light microscope (Nikon, Tokyo, Japan).

### Fatty acid analysis of the cells

LoVo and RKO cells seeded in 50mL cell culture flasks supplemented with various PUFAs and 5-FU for 48h were then harvested using trypsin. Subsequently, cells were washed twice with PBS, resuspended in 0.5 mL high purity water. The fatty acids were esterified as recommended by Seppänen-Laakso T [22], and Cellular content of fatty acids was measured by gas chromatography as described in detail previously [20]. The peaks were identified by comparing the retention times with known methyl ester standards (FAME Mix, Supelco) and the fatty acids were quantified by an external standard method.

### LTB_4_, PGE_2_ and LXA_4_ measurement

For the determination of LTB_4_, PGE_2_, and LXA_4_ levels in LoVo and RKO culture medium, cells were seeded in a 6-well plate for overnight and treated with pre-determined doses of PUFAs and 5-FU for 48h. At the end of the treatment period, the medium was harvested by centrifuging at 2,000×g for 5 min to remove floating cells. Subsequently, the supernatant was collected and assayed using LTB_4_, PGE_2_, and LXA_4_ ELISA kits following the manufacturer’s instructions (WESTANG Bio-TECH, Shanghai, China). The results were corrected by the protein level before expressing as percentage of the control.

### Determination of COX-2 and ALOX5 activity

LoVo and RKO cells were cultured in 6-well plates and treated with various PUFAs and 5-FU for 48h, at the end of which the cultural supernatant collected by centrifuging at 2,000×g for 5 min were used for COX-2 and ALOX5 levels analysis by competitive enzyme-linked immunosorbent assay (ELISA) purchased from Xitang (Shanghai, China) and CUSABIO (Wuhan, China), respectively. The results were corrected by the protein level before expressing the values as percentage of the control.

### mRNA analysis

Total RNA was extracted from cultured LoVo and RKO cells supplemented with various PUFAs and 5-FU for 48h with 500μL TRIzol reagent (Haogene Biotech, Hangzhou, China). cDNA was synthesized by a reverse transcription reaction with 1st-Strand cDNA Synthesis Kit (Haogene Biotech, Hangzhou, China) from 500ng of total RNA according to the manufacturer’s instructions. Real-time quantitative PCR (RT-PCR) was performed using Power SYBR Master Mix (Invitrogen). For each reaction, 12.5 ul of Power SYBR Master Mix, 0.5μl of each PCR primer, 1μl of DNA template, 10.5μl of distilled water were added. Human18S rRNA was chosen as a reference for gene expression analysis, and further analysed according to the 2^－ΔΔCT^ method.

Primers (Sangon Biotech) used were: Human 18s (F; GACTCAACACGGG-AAACCTCAC, R; CCAGACAAATCGCTCCACCAAC), Human PGDS (F; CCTGCCCCAAACCGATAAGTG, R; GCTCGGGGAAGGAACAGAGCAG), Human mPGES (F; CCCCAGTATTGCAGGAGCGAC, R; GCATCCAGGCGACA-AAAGGGTTAG).

### Statistical analysis

Each experiment was repeated at least three times and the data obtained was analysed with SAS 8.0 software and SPSS software version 16.0. The results are expressed as means ± SD. All data were analysed using the ANOVA procedure with significance analysis at *p* < 0.05 level.

## Results

### PUFAs induced morphological changes in LoVo and RKO cells

**Fig 1:**
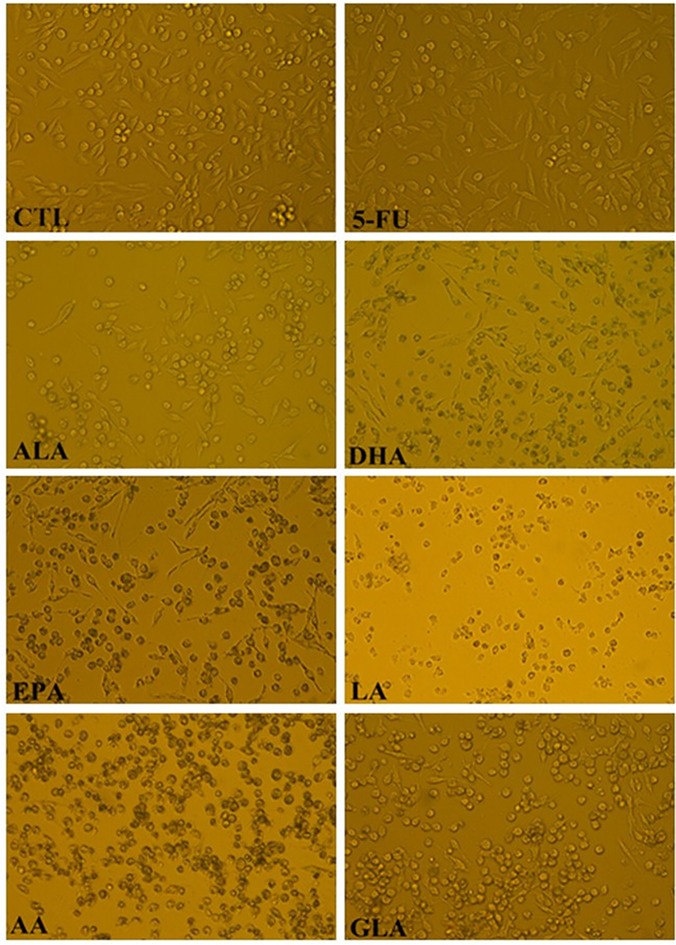
The cell morphological changes of LoVo cells were observed by inverted light microscopy (100×) after treated with ALA (150μM), EPA (150μM), DHA (150μM), LA (150μM), GLA (300μM), AA (150μM), 5-FU (10μM) for 48h.

**Fig 2:**
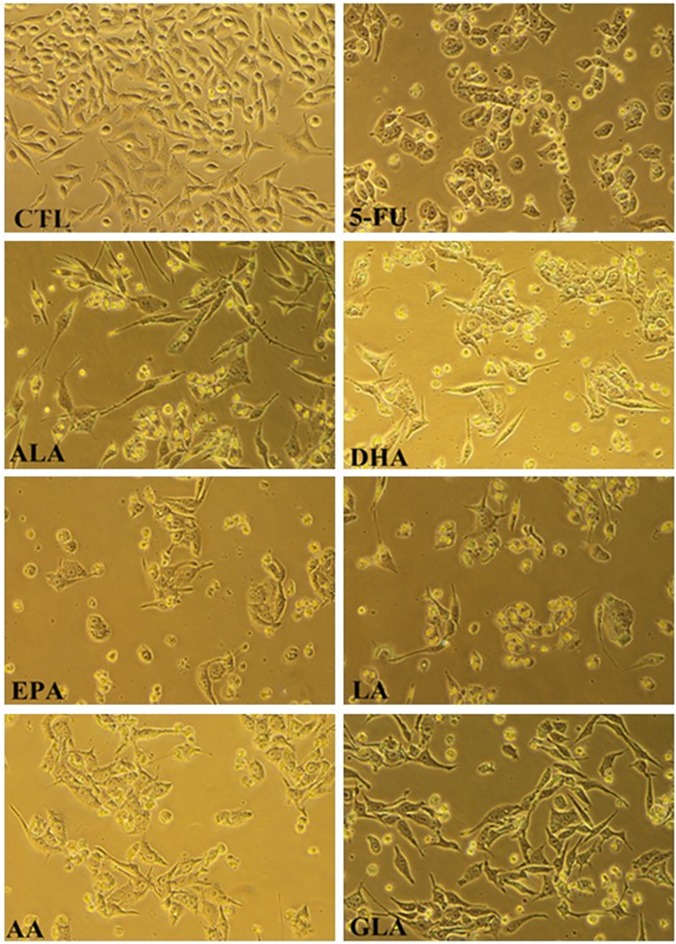
The cell morphological changes of RKO cells after treatment with ALA (140μM), EPA (120μM), DHA (120μM), LA (120μM), GLA (200μM), AA (120μM), 5-FU (0.4 μM) for 48 h.

The morphological changes of LoVo and RKO cells in response to different doses of PUFAs and 5-FU treatment were analyzed under light microscopy and the results are shown in Figs 1 and 2. The results showed that untreated cells grew well with a normal shape, which could be found by their uniform distribution with intensive connections and confluence. However, compared with the control group, PUFAs and 5-FU treatment of LoVo and RKO cells decreased their number, and cell shape was distorted with rounding up, which suggests that PUFAs and 5-FU inhibited the proliferation of these cells.

### Effect of PUFAs on the fatty acids composition of colon cancer cells

**Fig 3:**
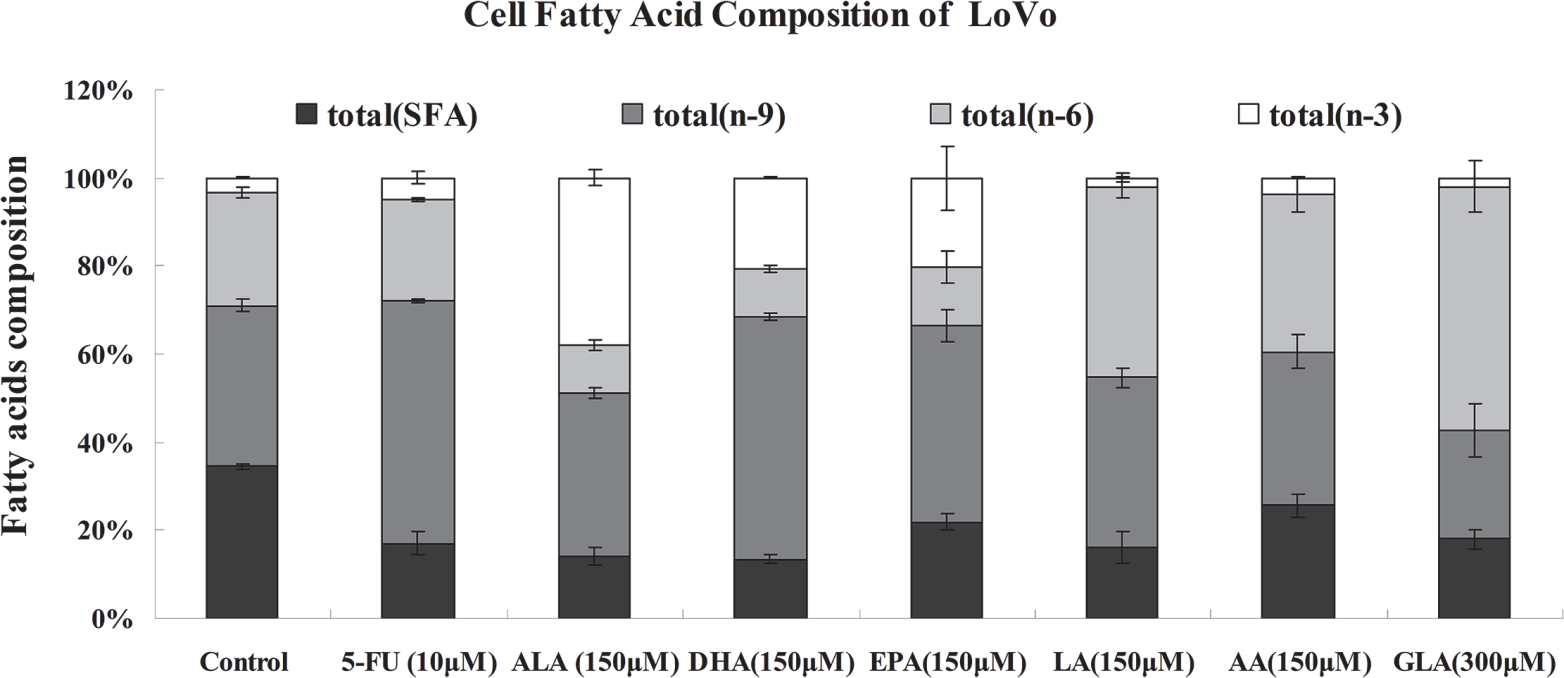
Changes in the fatty acid compositions of LoVo cells that were supplemented for 48 hours with various PUFAs and 5-FU.

**Fig 4:**
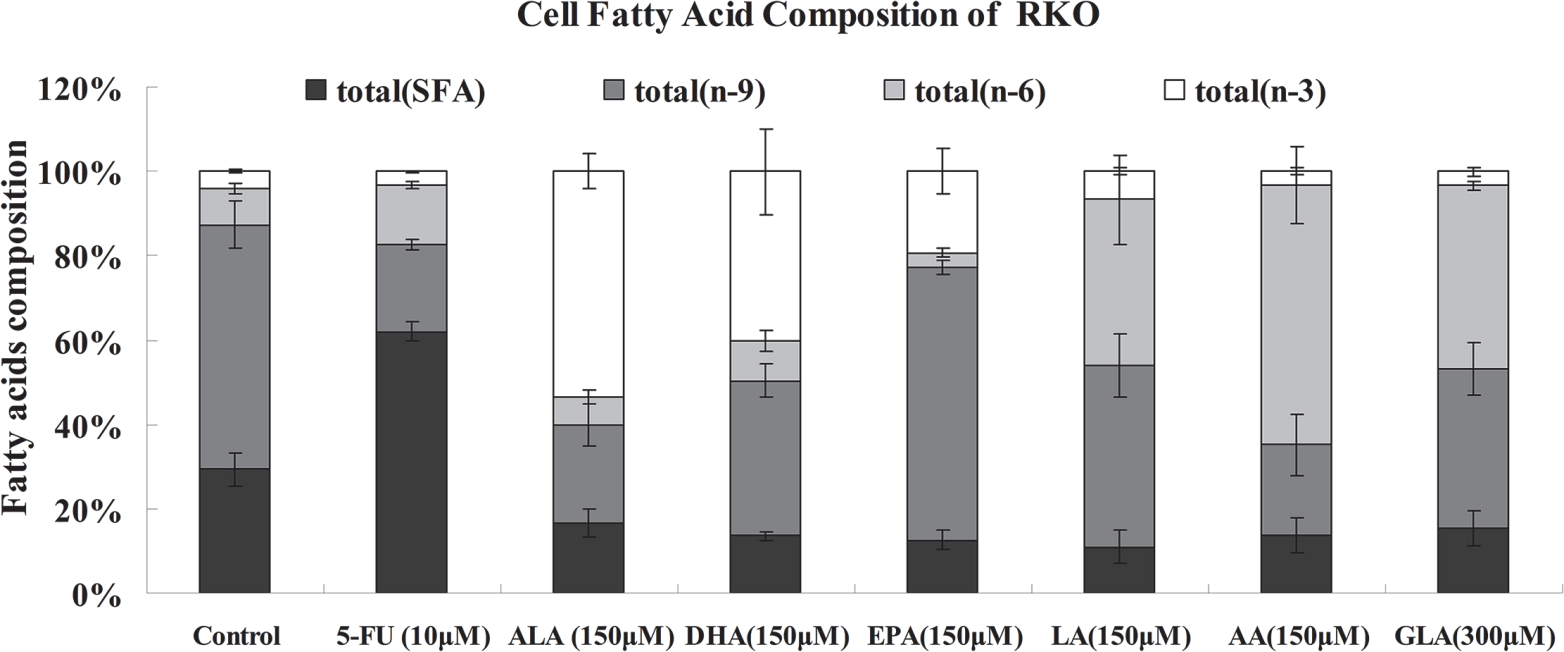
Changes in the fatty acid compositions of RKO cells that were supplemented for 48 hours with various PUFAs and 5-FU.

In the present study, we analyzed fatty acid composition of LoVo and RKO cells in response to supplementation with various PUFAs and 5-FU for 48h and are shown in Table 1 and Figs 3 and 4. The ratio of unsaturated fatty acids/saturated fatty acids (S/U) and the ratio of n-6 PUFAs/n-3 PUFAs (n-6/n-3) were also calculated. These results indicated that when supplemented with PUFAs, fatty acid profiles of LoVo and RKO cells were significantly different compared with control group. As shown in Table 1, it is noteworthy that levels of supplemented fatty acids were significantly increased in LoVo and RKO cells. For instance, LoVo cells supplemented with ALA, DHA, EPA, LA, AA, GLA showed a 54.9-fold, 12.32-fold, 16.6-fold, 13.95-fold, 3.17-fold and 39.88-fold higher concentrations respectively compared to the control. On the other hand, RKO cells treated with ALA, DHA, EPA, LA, AA, GLA showed a 14.09-fold, 54.81-fold, 225.95-fold, 31.71-fold, 44.46-fold and 18.41-fold increase in the concentrations of these fatty acids respectively. It is noteworthy that both in LoVo and RKO cells showed a substantial increase in EPA and DHA levels when supplemented with EPA suggesting that EPA is converted to its long-chain metabolite DHA. In addition, supplementation of AA enhanced the content of AA and EPA in both LoVo and RKO cells. Surprisingly, LoVo cells supplemented with GLA resulted in not only a significant increase in their GLA content but also an increase in ALA, EPA, DHA, LA and AA. In contrast, RKO cells supplemented with GLA showed a decrease in ALA, EPA, DHA and LA content in comparison to control.

**Table 1:** Fatty acid analysis of LoVo and RKO cells in response to supplementation of various PUFAs and 5-FU for 48h.

**LoVo cells**	**Control**	**5-FU(10μM)**	**ALA (150μM)**	**DHA(150μM)**	**EPA(150μM)**	**LA(150μM)**	**AA(150μM)**	**GLA(300μM)**
**ALA**	1.00±0.18	2.10±0.19**	54.9±7.42***	1.11±0.2	1.79±0.57	2.33±0.42	N.D	1.5±0.4
**EPA**	1.00±0.34	1.47±0.08	1.9±0.21	1.43±0.24	16.6±0.73***	1.08±0.17	3.2±0.6*	1.96±0.71*
**DHA**	1.00±0.04	0.71±0.01**	0.86±0.07	12.32±2.47*	8.65±1.46*	0.98±0.11	0.66±0.07*	1.75±0.61
**LA**	1.00±0.09	0.60±0.01*	0.61±0.13	0.58±0.13	0.87±0.01	13.95±0.66**	0.18±0.04**	1.26±0.46
**AA**	1.00±0.13	0.69±0.0*	0.81±0.09	0.71±0.14	0.67±0.04*	2.46±0.15*	3.17±0.11*	2.32±0.91
**GLA**	1.00±0.15	1.68±0.07**	0.57±0.11*	1.8±0.46	1.01±0.36	1.11±0.21	1.83±0.11	39.88±5.73**
**U/S**	1.90±0.05	4.86±0.09**	6.14±0.10**	6.52±0.58*	3.59±0.41	5.41±1.41	2.93±0.39	4.62±0.68
**n-6/n-3**	7.53±0.91	4.78±1.44	0.29±0.03*	0.53±0.09*	0.72±0.14*	21.19±5.29	11.16±3.20	28.33±1.67*
**RKO cells**	**Control**	**5-FU(0.4 μM)**	**ALA(140μM)**	**DHA(120μM)**	**EPA (120μM)**	**LA (120μM)**	**AA (120μM)**	**GLA(200μM)**
**ALA**	1.00±0.10	0.42±0.02*	14.09±2.01**	1.01±0.17	1.34±0.14	2.48±0.67	1.13±0.42	0.68±0.03*
**EPA**	1.00±0.07	2.13±0.11**	2.21±0.66	3.68±1.08	225.95±33.45**	1.90±0.28*	2.40±0.24**	0.50±0.09*
**DHA**	1.00±0.13	N.D	2.05±0.31*	54.81±8.36**	17.92±6.21*	3.07±0.81*	0.87±0.19	0.69±0.32
**LA**	1.00±0.12	0.42±0.02*	0.68±0.23	0.58±0.31	1.78±0.24**	31.71±3.00**	0.78±0.12	0.49±0.11**
**AA**	1.00±0.07	1.22±0.06	1.63±0.29*	1.36±0.34	4.20±0.78*	2.58±1.75	44.46±7.22**	2.13±0.89
**GLA**	1.00±0.15	1.03±0.05	0.65±0.09*	0.58±0.30	1.39±0.39	N.D	2.96±1.45	18.41±3.49*
**U/S**	2.44±0.44	0.61±0.06*	4.05±0.41*	6.34±0.52*	10.40±3.37	6.35±1.95	6.79±1.57	5.92±1.28
**n-6/n-3**	1.99±0.14	4.31±0.08***	0.12±0.02***	0.29±0.09**	0.13±0.03***	17.21±6.79	20.94±3.98*	13.98±2.71*

U/S: the ratio of unsaturated fatty acids/saturated fatty acids; n-6/n-3: the ratio of n-6 PUFAs/n-3 PUFAs. The basal value (control) was taken as 1.00,

*P < 0.05,

**P < 0.01,

***P < 0.001 compared to control.

While assessing the ratio between unsaturated fatty acids/saturated fatty acids (U/S), it was noted that both LoVo and RKO cells showed a higher ratio of U/S in all PUFAs treatments compared to the control as is evident form the results shown in Table 1 and Figs 3 and 4. LoVo cells supplemented with n-6 PUFAs (LA, AA and GLA) resulted in a 17.52, 10.09 and 29.68% increases for the sum of n-6 PUFAs and 1.13, 0.1, 1.46% decreases for the sum of n-3 PUFAs compared to control. Similarly, supplementation with n-3 PUFAs (ALA, DHA and EPA) to LoVo cells resulted in a 34.52, 17.22, 16.83% increases in the total amount of n-3 PUFAs and 14.78, 14.73, 12.12% decreases for the sum of n-6 PUFAs in comparison with control. RKO cells that were supplemented with LA, AA and GLA showed a 30.69, 53.33 and 35.84% increase in the concentrations of n-6 PUFAs compared to the control respectively. On the other hand, RKO cells that were supplemented with ALA, DHA and EPA showed an increase in the levels of these n-3 fatty acids by 49.21, 35.83 and 15.18% respectively compared to the control.

Supplementation of 5-FU to LoVo and RKO cells produced far few changes in the composition of n-6 PUFAs and n-3 PUFAs in comparison with control. On the other hand, LoVo cells showed a significant increase in the content n-9 PUFAs and decreased content of saturated fatty acids. In contrast, supplementation of 5FU produced a significant increase in the saturated fatty acid content and a significant decrease in its n-9 PUFAs in RKO cells.

### Effects of PUFAs on the PGE_2_, LTB_4_ and LXA_4_ level

PGE_2_ and LTB_4_ have been shown to posses pro-inflammatory actions, promote tumor cell invasion, enhance their metastasis, upregulate VEGF expression, inhibit tumor cell apoptosis and suppress immune function [13, 23]. Thus, both PGE_2_ and LTB_4_ may act as anti-apoptotic molecules and enhance tumor growth. In contrast, lipoxin A_4_ is a potent anti-inflammatory molecule and opposes the actions of PGE_2_ and thus, could suppress tumor cell proliferation, invasiveness and metastasis [13]. In view of this, we determined the secretion of PGE_2_, LTB_4_ and LXA_4_ by LoVo and RKO cells following supplementation with various PUFAs and 5-FU for 48 hours.

**Fig 5:**
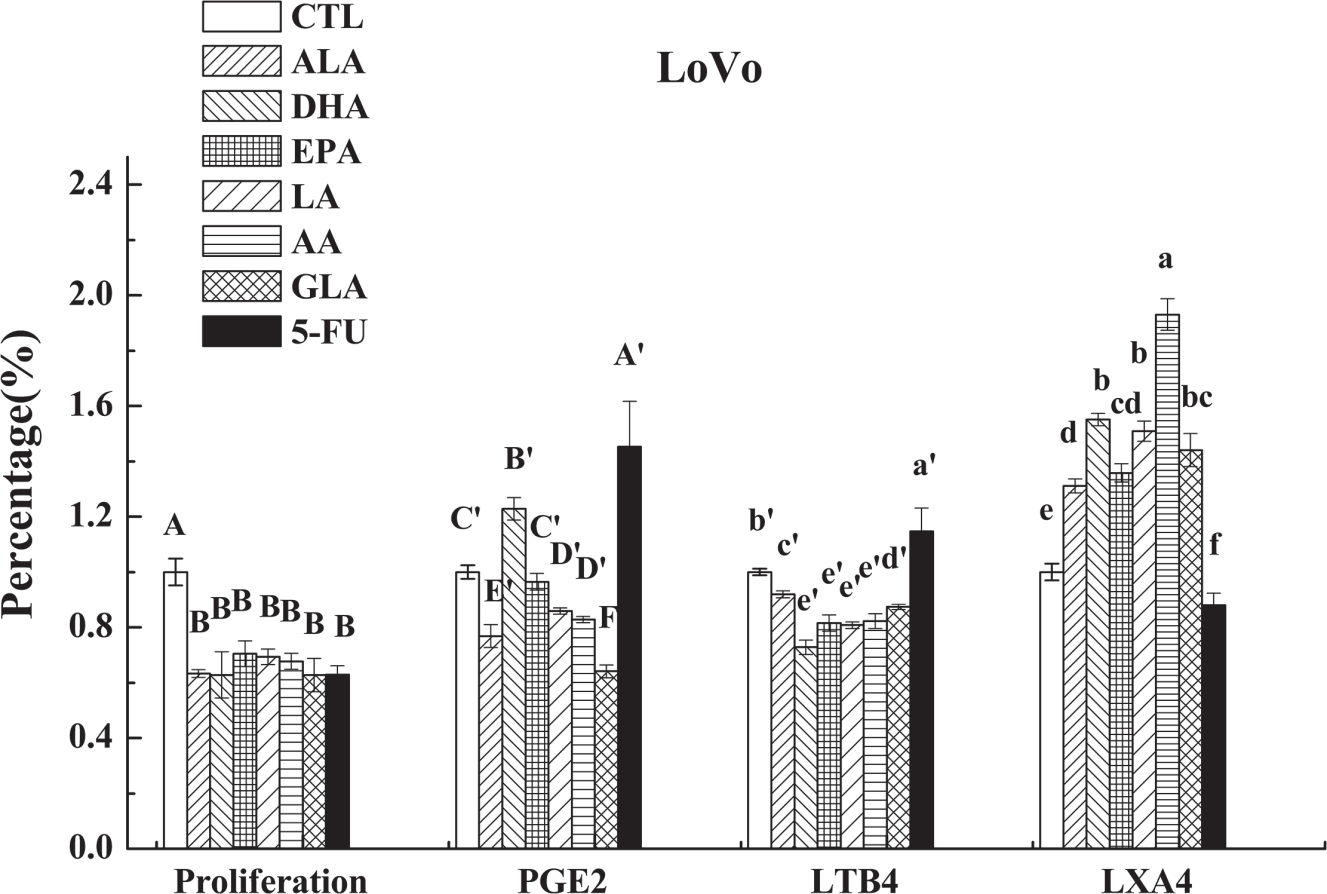
Effect of ALA, DHA, EPA, LA, AA, GLA and 5-FU on the expression of PGE_2_, LTB_4_ and LXA_4_ in LoVo cells by ELISA. Values in the same column with different letters are significantly different (p<0.05).

**Fig 6:**
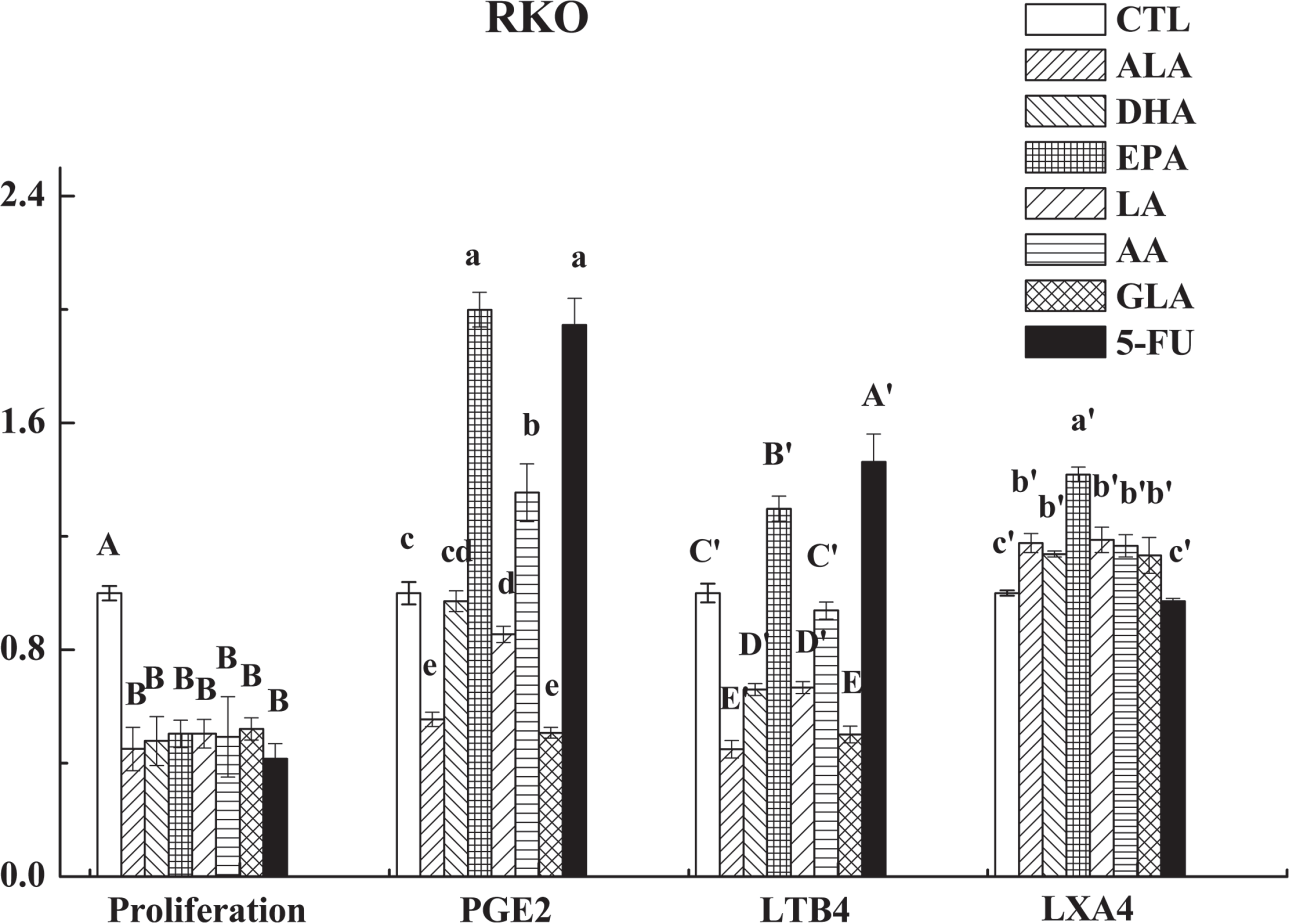
Effect of ALA, DHA, EPA, LA, AA, GLA and 5-FU on the expression of PGE_2_, LTB_4_ and LXA_4_ in RKO cells by ELISA. Values in the same column with different letters are significantly different (p<0.05).

As is evident from these results shown in Fig 5, PGE_2_ secretion by LoVo cells when supplemented with LA, GLA, AA, ALA, EPA and 5-FU was suppressed, while DHA enhanced its secretion compared with the control. On the other hand, LA, GLA, AA, ALA, EPA and DHA (all PUFAs tested) decreased LTB_4_ secretion to a significant degree by LoVo cells while 5-FU enhanced the same. Results shown in Fig 6 revealed that RKO cells supplemented with LA, GLA, DHA and ALA produced decreased amounts of PGE_2_, while AA, EPA and 5-FU induced a substantial increase in its production. In contrast, RKO cells showed decreased production of LTB_4_ when supplemented with LA, GLA, AA, ALA and DHA and showed increased production in response to EPA and 5-FU challenge.

**Fig 7:**
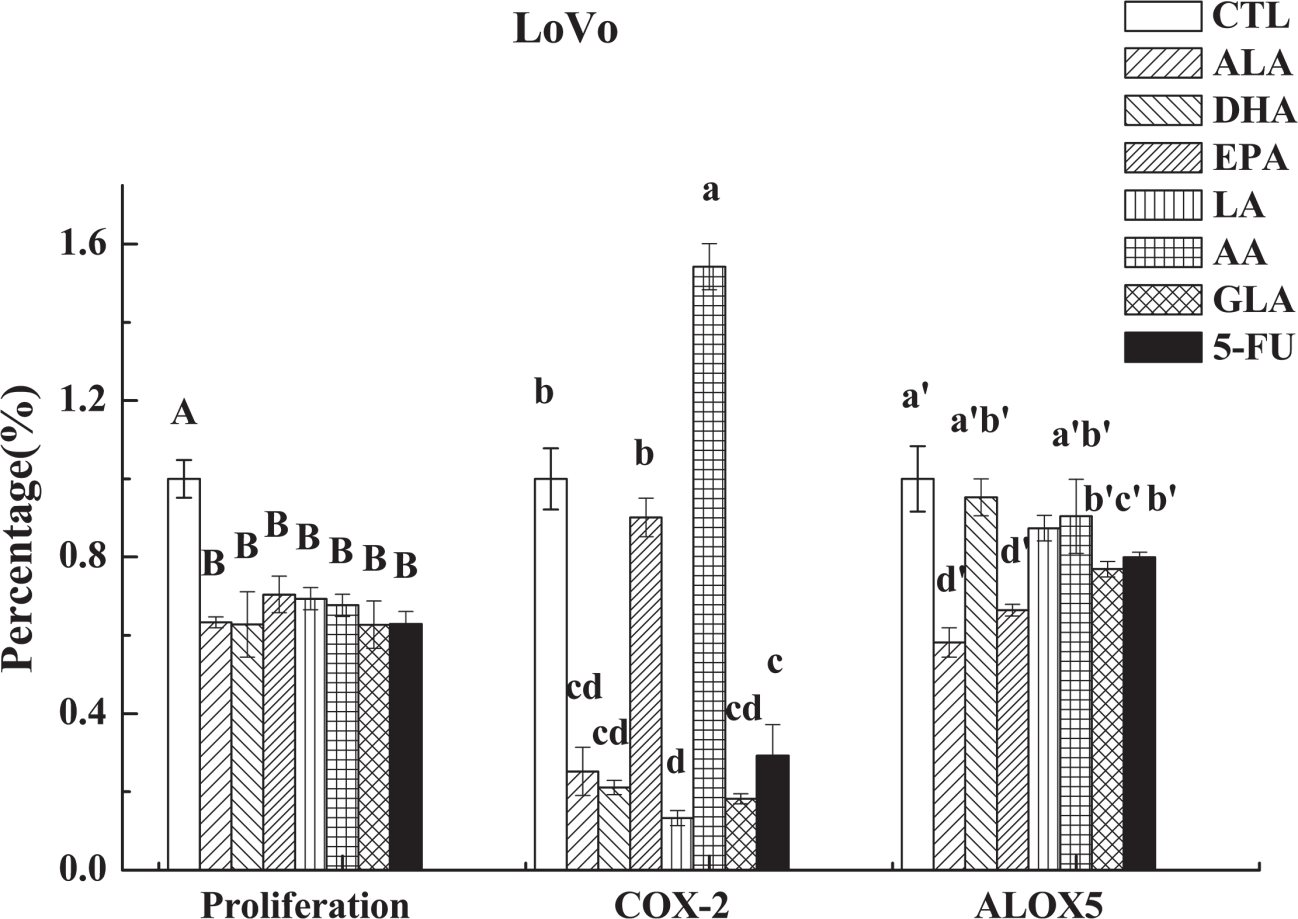
Effect of ALA, DHA, EPA, LA, AA, GLA and 5-FU on the expression of COX-2 and ALOX5 in LoVo cells by ELISA. Values in the same column with different letters are significantly different (p<0.05).

In comparison to these results, all PUFAs tested enhanced the production of LXA_4_ by LoVo and RKO cells while, 5-FU treatment inhibited LXA_4_ secretion in both these cells as shown in Figs 6 and 7.

### Effects of PUFAs on the expression of COX-2 and ALOX5

**Fig 8:**
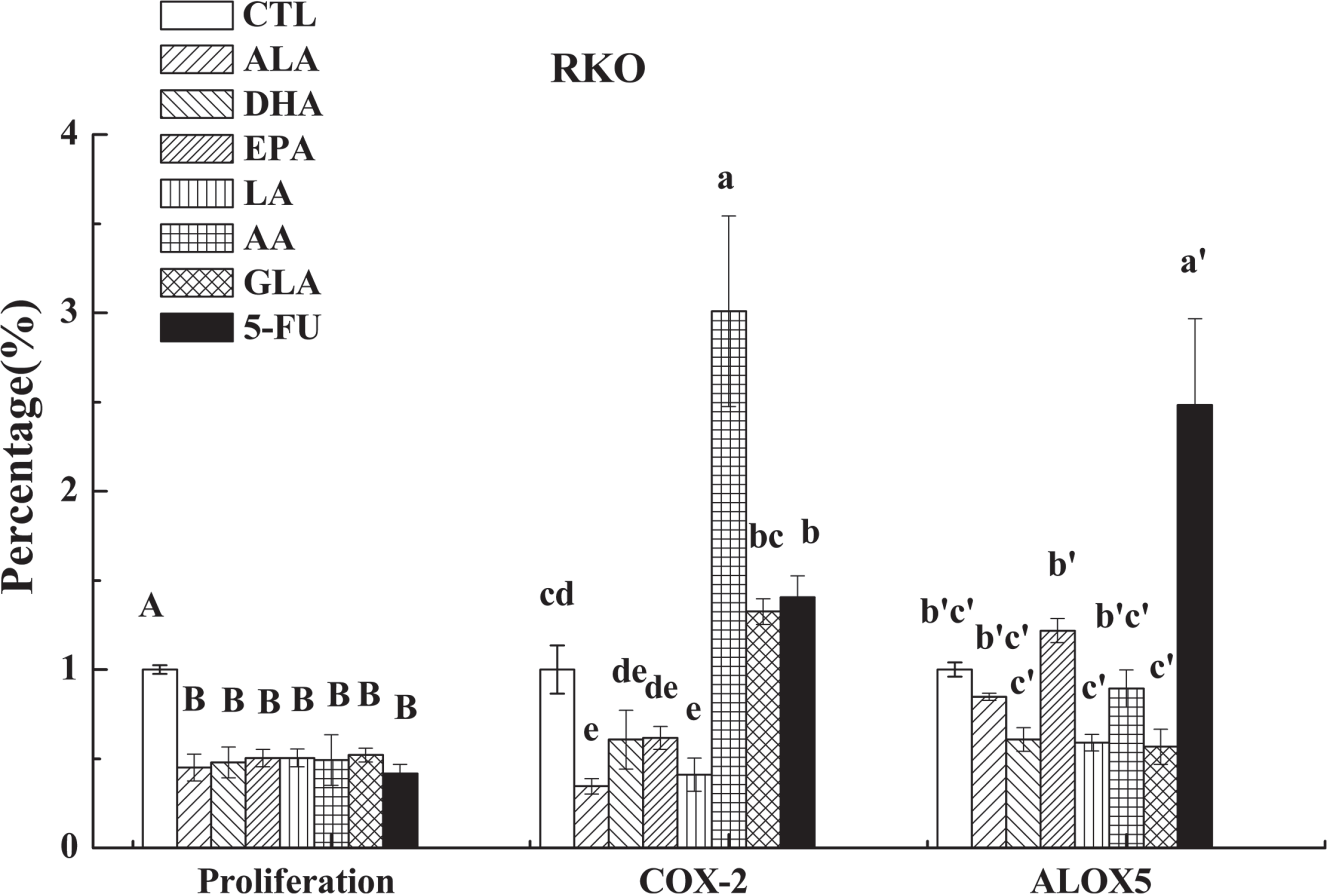
Effect of ALA, DHA, EPA, LA, AA, GLA and 5-FU on the expression of COX-2 and ALOX5 in RKO cells by ELISA. Values in the same column with different letters are significantly different (p<0.05).

COX-2 plays an important role in both inflammation and colon carcinogenesis and hence, it has been suggested that agents that suppress its expression may be useful in inhibiting these two processes [24]. On the other hand, growing body of evidence indicates that arachidonate 5-lipoxygenase (ALOX5) is a crucial regulator of inflammation and cancer pathogenesis [25]. In the present study, we measured the expression of COX-2 following supplementation of PUFAs and 5-FU, which showed that, in general, PUFAs (except for AA on LoVo and GLA and AA on RKO cells) down-regulated the expression of COX-2 compared to control as is evident from the results shown in Figs 6 and 7, while PUFAs (except for EPA on RKO cells) down-regulated the expression of ALOX5 compared to control as seen from the results depicted in Figs 7 and 8. On the other hand, 5-FU inhibited the expression of COX-2 and ALOX5 in LoVo cells but enhanced in RKO cells.

### Effects of PUFAs on the expression of PGDS and mPGES

Microsomal prostaglandin E synthase (mPGES) and PGD synthase (PGDS) mediate the final regulatory step of PGE_2_ and prostaglandin D_2_ biosynthesis respectively and have been implicated in tumor pathogenesis [26, 27]. Hence, we investigated the expression of PGE synthase and PGD synthase using real-time PCR following supplementation of PUFAs and 5-FU. Results of this study shown in Table 2 indicated that PGE_2_ synthase expression in LoVo was increased compared to the control group, while PUFAs (except for EPA and GLA on LoVo) up-regulated the expression of PGD synthase compared to control. Additionally, neither the expression of PGE_2_ synthase nor PGD synthase was detected in RKO cells because of their low expression in these cells.

**Table 2:** Effect of ALA, DHA, EPA, LA, AA, GLA and 5-FU on the expression of PGDS and mPGES in RKO cells by ELISA.

	LoVo Cells		RKO cells	
Fatty acid	mPGES	PGDS	mPGES	PGDS
Control	1.00±0.15 ^b^	1.00±0.12^c^ ^d^	N/D	N/D
LA	0.77±0.07^c^ ^d^	1.90±0.09 ^a^	N/D	N/D
GLA	0.72±0.04^c^ ^d^	0.66±0.05 ^e^	N/D	N/D
AA	0.96±0.05 ^b^ ^c^	1.54±0.14^b^	N/D	N/D
ALA	0.63±0.07 ^c^ ^d^	1.04±0.08 ^c^ ^d^	N/D	N/D
EPA	0.58±0.04 ^d^	0.21±0.02 ^f^	N/D	N/D
DHA	0.94±0.10^b^ ^c^	1.33±0.10^b^ ^c^	N/D	N/D
5-FU	4.38±0.29 ^a^	0.94±0.12^d^ ^e^	N/D	N/D

N/D = Below the detectable limits.

Values in the same column with different letters are significantly different (p<0.05).

a P < 0.05 compared to respective controls or fatty acid/5-FU treatments.

b P < 0.05 compared to respective controls or fatty acid/5-FU treatments.

c P < 0.05 compared to respective controls or fatty acid/5-FU treatments.

d P < 0.05 compared to respective controls or fatty acid/5-FU treatments.

e P < 0.05 compared to respective controls or fatty acid/5-FU treatments.

f Significant difference (decreased) between EPA treatment and control with regard to PGDS.

## Discussion

There is evidence to suggest that PUFAs, especially, EPA and DHA have anti-cancer activity both *in vitro* and *in vivo*. Previously, we reported that PUFAs have substantial cytotoxic action on tumor cells by enhancing free radical generation, lipid peroxidation, altering membrane composition and inducing mitochondrial dysfunction [2, 3, 20, 28]. In an extension of this study, we now evaluated whether supplementation of PUFAs could produce changes in the formation of their metabolites that may have a role in bringing about their growth inhibitory action. The changes in the morphological appearance, fatty acid composition, PGE_2_, LTB_4_ and LXA_4_ secretion and COX-2, ALOX5, mPGES, PGDS expression noted in LoVo and RKO cells in response to supplementation of different PUFAs indicates that there could occur substantial changes in the way these fatty acids are metabolized by cancer cells that could account for their tumoricidal action. It is noteworthy that semi-differentiated colon cancer RKO cells are more sensitive to the actions of PUFAs and 5-FU than undifferentiated colon LoVo cancer cells.

Tumor cells are known to be deficient in the activity of Δ^6^ and Δ^5^ desaturases [29, 30] and thus, may contain substantially lower amounts of GLA, AA, EPA and DHA compared to normal cells. The exact reason for the low activity of desaturases in cancer cells is not known. Since PUFAs can undergo peroxidation easily and generate free radicals that are toxic to cells [2, 3], it is likely decreased activity of desaturases is a defence mechanism adopted by tumor cells to protect themselves from these toxic molecules. As expected, supplemented fatty acids suppressed the proliferation of LoVo and RKO cells due to their incorporation in to the cell membrane to a significant degree. But, what is surprising is the observation that supplementation of AA enhanced EPA content to a significant degree both in LoVo and RKO cells suggesting that a close interaction exists between n-3 and n-6 fatty acids [9, 20]. Previously, none described the existence of such an interaction between n-3 and n-6 PUFAs influencing the formation of PGs, LTs, and other metabolites. In fact, previous studies showed that supplementation of EPA and DHA decreased AA content in the cell membrane lipid pool of tumor cells [14–16, 23].

**Fig 9:**
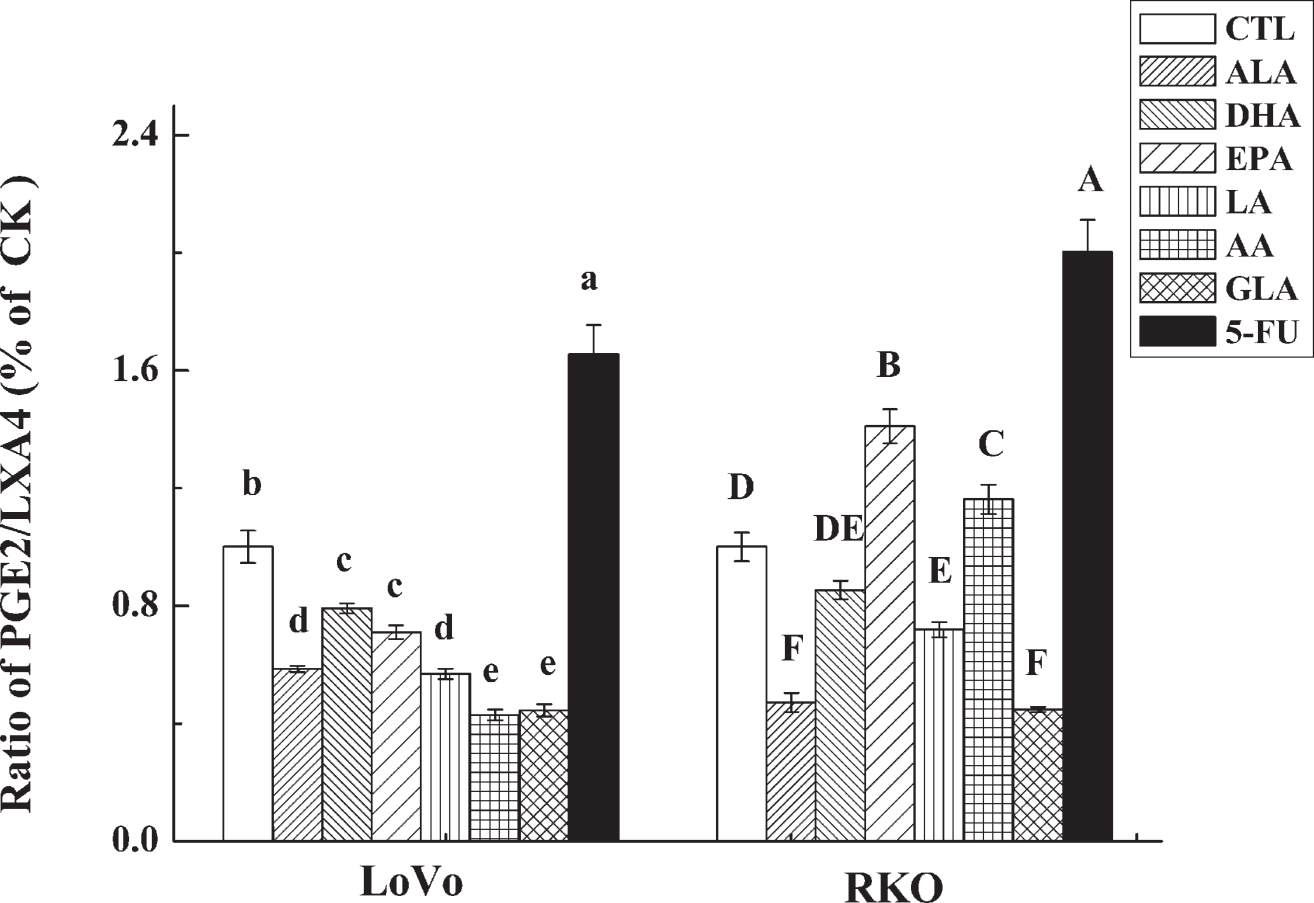
Effect of ALA, DHA, EPA, LA, AA, GLA and 5-FU on the expression of PGE_2_/LXA_4_ in LoVo and RKO cells in vitro. Values in the same column with different letters are significantly different (p<0.05).

AA, EPA and DHA form precursors not only to pro-inflammatory molecules PGs, TXs and LTs but also give rise to potent anti-inflammatory molecules LXs, resolvins, protectins, maresins and nitrolipids [9, 31, 32]. PGE_2_ and PGF_2α_ and LTA_4_ and LTB_4_ are known to be produced in significantly higher amounts by tumor cells, which may enhance their motility and invasive capacity [33]. In contrast, LXA_4_ may promote apoptosis and inhibit growth of tumors by suppressing tumor angiogenesis by inhibiting the production of VEGF [34]. This suggests that balance between pro-inflammatory PGs, LTs and TXs and anti-inflammatory LXs, resolvins, protectins, maresins and nitrolipids (that are formed due to reaction between various PUFAs and nitric oxide and these compounds have anti-inflammatory actions) may determine the degree of proliferation and invasive capacity of tumor cells [8]. In addition, PGD_2_ can also inhibit cell proliferation and induce apoptosis in various cell types, including colon cancer cells [35]. In the present study, the expression of PGD synthase (a rate limiting enzyme that regulates the synthesis of prostaglandin D2) was found to be up-regulated by PUFAs (except for EPA and GLA) compared to control in LoVo. On the other hand, it was observed that majority of the PUFAs except DHA inhibited the release of PGE_2_, while all PUFAs inhibited LTB_4_ production but enhanced the synthesis and release of LXA_4_ in LoVo cells. Similarly, all PUFAs except AA and EPA inhibited PGE_2_ and LTB_4_ (except EPA) but enhanced the production of LXA_4_ in RKO cells (see Figs 5 and 6). Furthermore, it is noteworthy that DHA, EPA and AA induced significant increase in the secretion of LXA_4_ compared to other PUFAs suggesting that anticancer actions of these three PUFAs may due to their ability to enhance the formation of LXA_4_ in addition to suppressing PGE_2_ and LTB_4_ synthesis_._ This is evident from the data shown in Fig 9 wherein we calculated the PGE_2_/LXA_4_ ratio. All PUFAs reduced this ratio in LoVo cells compared to control except 5-FU, whereas AA, EPA (AA > EPA) and 5-FU increased this ratio in RKO cells. Thus, in general, all PUFAs enhanced the formation of LXA_4_ and decreased the synthesis of PGE_2_ and LTB_4_ tilting the balance more towards anti-inflammatory status. (Fig 9: Effect of ALA, DHA, EPA, LA, AA, GLA and 5-FU on the expression of PGE_2_/LXA_4_ in LoVo and RKO cells in vitro; Values in the same column with different letters are significantly different (p<0.05). A summary of the data obtained in the present study showing the changes that occurred as a result of the action of various PUFAs and 5-FU on the secretion of PGE_2_, LTB_4_, LXA_4,_ ALOX5, COX-2, PGDS and mPGES activity in LoVo and RKO is given in Table 3 for easy reference. (Table 3: Summary of the effect of various PUFAs and 5-FU on the secretion of PGE_2_, LTB_4_, LXA_4,_ ALOX5, COX-2, PGDS and mPGES activity in LoVo and RKO cells *in vitro*. N/D = Below detectable limits).

**Table 3:** Summary of the effect of various PUFAs and 5-FU on the secretion of PGE_2_, LTB_4_, LXA_4,_ ALOX5, COX-2, PGDS and mPGES activity in LoVo and RKO cells *in vitro*.

	LoVo Cells							RKO cells						
Fatty acid	PGE_2_	LXA_4_	LTB_4_	ALOX5	COX-2	mPGES	PGDS	PGE_2_	LXA_4_	LTB_4_	ALOX5	COX-2	mPGES	PGDS
LA	**↓**	**↑**	**↓**	**↓**	**↓**	**↓**	**↑↑**	**↓**	**↑**	**↓**	**↓**	**↓**	N/D	N/D
GLA	**↓**	**↑**	**↓**	**↓**	**↓**	**↓**	**↓**	**↓**	**↑**	**↓**	**↓**	**↑**	N/D	N/D
AA	**↓**	**↑↑**	**↓**	**↓**	**↑↑**	**↓**	**↑**	**↑**	**↑**	**↓**	**↓**	**↑↑**	N/D	N/D
ALA	**↓**	**↑**	**↓**	**↓**	**↓**	**↓**	**↑**	**↓**	**↑**	**↓**	**↓**	**↓**	N/D	N/D
EPA	**↓**	**↑**	**↓**	**↓**	**↓**	**↓**	**↓↓**	**↑↑**	**↑**	**↑**	**↑**	**↓**	N/D	N/D
DHA	**↑**	**↑↑**	**↓**	**↓**	**↓**	**↓**	**↑**	**↓**	**↑**	**↓**	**↓**	**↓**	N/D	N/D
5-FU	**↑**	**↓**	**↑**	**↓**	**↓**	**↑↑**	**↓**	**↑↑**	**↓**	**↑**	**↑↑**	**↑**	N/D	N/D

N/D = Below detectable limits.

COX-2 is needed for the synthesis of PGs, LTs, TXs and LXs. Several studies reported that COX-2 expression is elevated in colorectal tumors in comparison to normal colorectal tissue [36]. Our results reported here indicated that all PUFAs except AA (in LoVo) and GLA and AA (in RKO) suppressed the expression of COX-2. This is consistent with the results that PGE_2_ and LTB_4_ secretion is inhibited by these PUFAs that may, in turn, inhibit tumor cell proliferation. It may be mentioned here that COX-2 is also needed for the synthesis of LXA_4_ that may explain why AA enhanced its (COX-2) activity in LoVo and RKO cells that corresponded to the enhanced production of LXA_4_ seen in these cells. It is noteworthy that the secretion of PGE_2_ and LTB_4_ corresponded with the activity of mPGES and ALOX5, which indicates that upregulated activity of mPGES and ALOX5 could be responsible for the enhanced secretion of PGE_2_ and LTB_4_ seen in the LoVo and RKO cells.

For the first time, we showed that the growth inhibitory action of PUFAs is due to an increase in the production of LXA_4_ in colon cancer cells. Previously, several studies showed that EPA and DHA suppress the proliferation of tumor cells by inhibiting the expression of COX-2. In contrast to these studies, we noted in the present study that AA suppressed the growth of colon cancer cells and at the same time upregulated that of COX-2 expression yet enhanced the production of LXA_4_ that explains its growth suppressive action. Hence, we suggest that decrease in the production of PGE_2_, leukotriene B_4_ and an increase in LXA_4_ is more crucial for the growth inhibitory actions of PUFAs rather than suppression of COX-2.

It is noteworthy that though all the PUFAs tested and 5-FU induced same degree of growth inhibition of both LoVo and RKO cells, changes observed in the secretion of PGE_2_, LTB_4_ and LXA_4_ and COX-2 expression were found to be variable. Despite this, certain generalizations are possible. Low amounts of AA, EPA and DHA in tumor cells, due to decreased activity of Δ^6^ and Δ^5^ desaturases, may trigger to augment PGE_2_ synthesis as a compensatory mechanism [37]. This is supported by the observation that plasma and/or other body fluid PGE_2_ levels are increased in inflammatory conditions lupus, rheumatoid arthritis and multiple sclerosis [38–40] (and cancer is also an inflammatory condition) in which deficiency of PUFAs has been described [41, 42]. On the other hand, oral administration of AA and DHA to animals with inflammatory colitis showed enhanced LXA_4_ synthesis with little or no change in PGE_2_ formation in AA supplemented animals while DHA decreased PGE_2_ formation with no change in LXA_4_ [43]. These results suggest that when AA, EPA and DHA levels are low, administration of these fatty acids augments the production of LXA_4_ (and possibly that of other anti-inflammatory molecules such as resolvins, protectins and maresins) with marginal changes in PGE_2_ synthesis. In addition, LXA_4_ is known to suppress PGE_2_ synthesis [44, 45]. It is likely that enhanced LXA_4_ production outweighs the changes in PGE_2_ levels such that ultimately inflammatory process is suppressed. Since LXA_4_ inhibited the growth of LoVo and RKO tumor cells [29, 46], enhanced the production of LXA_4_ on supplementation of AA, EPA and DHA and other PUFAs, it is likely that augmented formation of LXA_4_ (in addition to decreased formation of PGE_2_ and LTB_4_) could be responsible for the decreased growth of LoVo and RKO cells noted in the present study.

In conclusion, our findings indicate that PUFAs induce toxicity at 48h of colon cancer LoVo and RKO cells *in vitro*. Cytotoxic action of PUFAs may be associated with decrease in the production of pro-inflammatory PGE_2_ and LTB_4_, COX-2, mPGES and ALOX5 expression and enhanced formation of anti-inflammatory LXA_4_ (Table 2). These results are supported by our recent data that showed that lipoxins, resolvins and protectins have a direct growth inhibitory action on tumor cells [45, 46]. One important question that arises when only *in vitro* studies are performed is how relevant are these test tube results in an *in vivo* situation. Previously, we did perform both *in vivo* experiments and limited clinical studies that did show that some of these PUFAs, especially GLA, have tumoricidal actions against a variety of tumors [47–50]. Given the tumoricidal action of various PUFAs, it is important to dissect the mechanisms of their action. Since such studies are not always possible in an *in vivo* situation and humans, we have resorted to the present study to know the metabolism of PUFAs and the expression of mPGES, COX-2 and ALOX5 are altered in colon cancer cells. It is also true that all tumor cells many not behave in the same fashion (colon cancer cells may metabolize PUFAs differently compared to neuroblastoma cells) and the metabolism of different PUFAs may be different among several tumor cells. Nevertheless some generalizations may be possible. Thus, based on the results of the present study and our previous studies, we propose that tumor cell growth inhibitory or cytotoxic action of PUFAs is likely to be associated with decrease in the production of pro-inflammatory PGE_2_ and LTB_4_, COX-2, mPGES and ALOX5 expression and enhanced formation of anti-inflammatory lipoxins, resolvins and protectins. Further studies are being performed to verify these possibilities.
